# Hepatitis E genotype 3 virus isolate from wild boar is capable of replication in non-human primate and swine kidney cells and mouse neuroblastoma cells

**DOI:** 10.1186/s12917-020-02315-5

**Published:** 2020-03-21

**Authors:** Juozas Grigas, Evelina Simkute, Martynas Simanavicius, Arnoldas Pautienius, Zaneta Streimikyte-Mockeliune, Dainius Razukevicius, Arunas Stankevicius

**Affiliations:** 1 Faculty of Veterinary Medicine, Laboratory of Immunology, Department of Anatomy and Physiology, Lithuanian University of Health Sciences, Tilzes str. 18, Kaunas, Lithuania; 2 Vilnius University Life Sciences Centre, Institute of Biotechnology, Sauletekio al. 7, Vilnius, Lithuania; 3 Faculty of Veterinary Medicine, Institute of Microbiology and Virology, Lithuanian University of Health Sciences, Tilzes str. 18, Kaunas, Lithuania; 4 Faculty of Medicine, Lithuanian University of Health Sciences, A. Mickeviciaus str. 9, Kaunas, Lithuania

**Keywords:** Hepatitis E virus, Wild boar, Kidney cells, Neuroblastoma cells, Animal cell lines

## Abstract

**Background:**

Wild boar-derived hepatitis E (HEV) genotype 3 virus has been successfully isolated in cell lines of human origin only. Considering the zoonotic potential and possible extrahepatic localisation of genotype 3 strain, it is important to investigate the viability of cell lines of different animal and tissue origins. Therefore, the objective of the present study was to determine the permissiveness of non-human primate (MARC-145 and Vero) and swine (PK-15) cell lines of kidney origin, and a mouse neuroblastoma (Neuro-2a) cell line for isolation of wild boar-derived HEV genotype 3.

**Results:**

This study showed that MARC-145, PK-15, Neuro-2a and Vero cell lines were permissive to wild boar-derived HEV genotype 3 subtype 3i harbouring viral genome equivalents of 1.12 × 10^7^ copies/ml, 2.38 × 10^5^ copies/ml, 2.97 × 10^7^ copies/ml and 4.01 × 10^7^ copies/ml after five serial passages respectively. In all permissive cell lines, HEV was continuously recovered from growth medium between five and at least 28 days post-infection. Peak loads of HEV genome equivalents were observed on days 7, 12, 19 and 30 in MARC-145 (2.88 × 10^7^ copies/ml), Vero (4.23 × 10^6^ copies/ml), Neuro-2a (3.15 × 10^6^ copies/ml) and PK-15 (2.24 × 10^7^ copies/ml) cell lines respectively. In addition, successful virus isolation was confirmed by immunofluorescence assay targeting HEV capsid protein and sequencing of HEV isolate retrieved from cell cultures.

**Conclusions:**

This study showed that wild boar-derived HEV genotype 3 subtype 3i strain was capable of infecting cell lines of animal origin, including primate and porcine kidney cells (MARC-145, PK-15 and Vero), and mouse neuroblastoma cells (Neuro-2a), supporting the notion of the capacity of HEV genotype 3 to cross the species barrier and extra-hepatic localisation of the virus. These findings warrant further studies of tested cell lines to investigate their capacity as an efficient system for HEV propagation. HEV isolates from other wild animal hosts should be isolated on tested cell lines in order to generate more data on HEV transmission between wild animal populations and their role as sources of human infections.

## Background

Hepatitis E virus (HEV) is a single-stranded RNA-positive virus that belongs to the genus Orthohepevirus in the family Hepeviridae and is a causative agent of human and animal hepatitis E. Seven known genotypes have currently been identified, three of which (genotypes 3, 4 and 7) are zoonotic [1]. Domestic pigs and wild boars are known to be reservoirs for both genotypes 3 and 4, while deer and rabbits may serve as additional reservoirs for genotype 3. Both genotypes 3 and 4 are associated with cross-species transmission, which has been experimentally demonstrated [2, 3], including chronic cases of human hepatitis E in the United States and Europe with a possible zoonotic cause [4]. High genetic similarities between human and animal isolates, and the ability of animal-derived HEV strains to infect cell lines originating from human tissue indicate transmission of genotypes 3 and 4 from animal to human hosts. In turn, direct contact with animals or consumption of infected animal meat can result in successful human infection. Human HEV cases associated with the consumption of wild animal meat and direct contact with infected animals have been reported in a variety of European countries and Japan [5, 6]. Hunters and foresters have been identified as the main risk groups associated with HEV infections of wild animal origin.

HEV isolation in cell cultures has proven to be a complicated task, hindering further research on virus entry, replication cycle, virion assembly, release and cross-species transmission. To date, human hepatocarcinoma cell (PLC/PRF/5, HepG2/C3A and Huh-7) and human lung cancer cell (A549) lines have primarily been chosen for HEV isolation purposes [7–9]. Only a limited number of cell lines originating from non-human animal tissue have been employed for isolation of HEV genotypes 3 and 4. To date, the only successful isolation of wild boar-derived HEV genotypes 3 and 4 acquired in Japan was carried out in human A549 and PLC/PRF/5 cell lines [10]. Therefore, there is currently no information available on the capacity of HEV strains circulating in European wild boar populations to infect cell lines of animal or human origin. HEV is also known to manifest extra-hepatic localisation in infected individuals, suggesting the capacity of the virus to infect cell lines of different tissue origin. In particular, kidney cell lines originating from non-human primates (FRhK-4) and swine (IBRS-2) have proven to be sufficient for prolonged replication of HEV [11, 12]. At present, there have been no successful attempts to use animal cell lines originating from tissues other than liver or lungs for wild boar-derived HEV isolation.

Recently, findings about the association of HEV infection with neurological symptoms have been accumulating, suggesting possible HEV localisation in the nervous system and utilisation of nervous tissue cells for viral isolation. Both immortalised human neuronal-derived cells and primary neurons have been found to support HEV replication, assembly and release [13, 14]. To date, there is no data on the symptomatic expression of HEV localisation in nervous tissue in animals or isolation of non-human derived HEV strains in neuronal cell lines. The objective of this study was to determine the permissiveness of non-human primate (MARC-145 and Vero) and swine (PK-15) cell lines of kidney origin and a mouse neuroblastoma (Neuro-2a) cell line for the isolation of wild boar-derived HEV genotype 3.

## Results

### Phylogenetic analysis of the wild boar-derived HEV strain

**Fig. 1:**
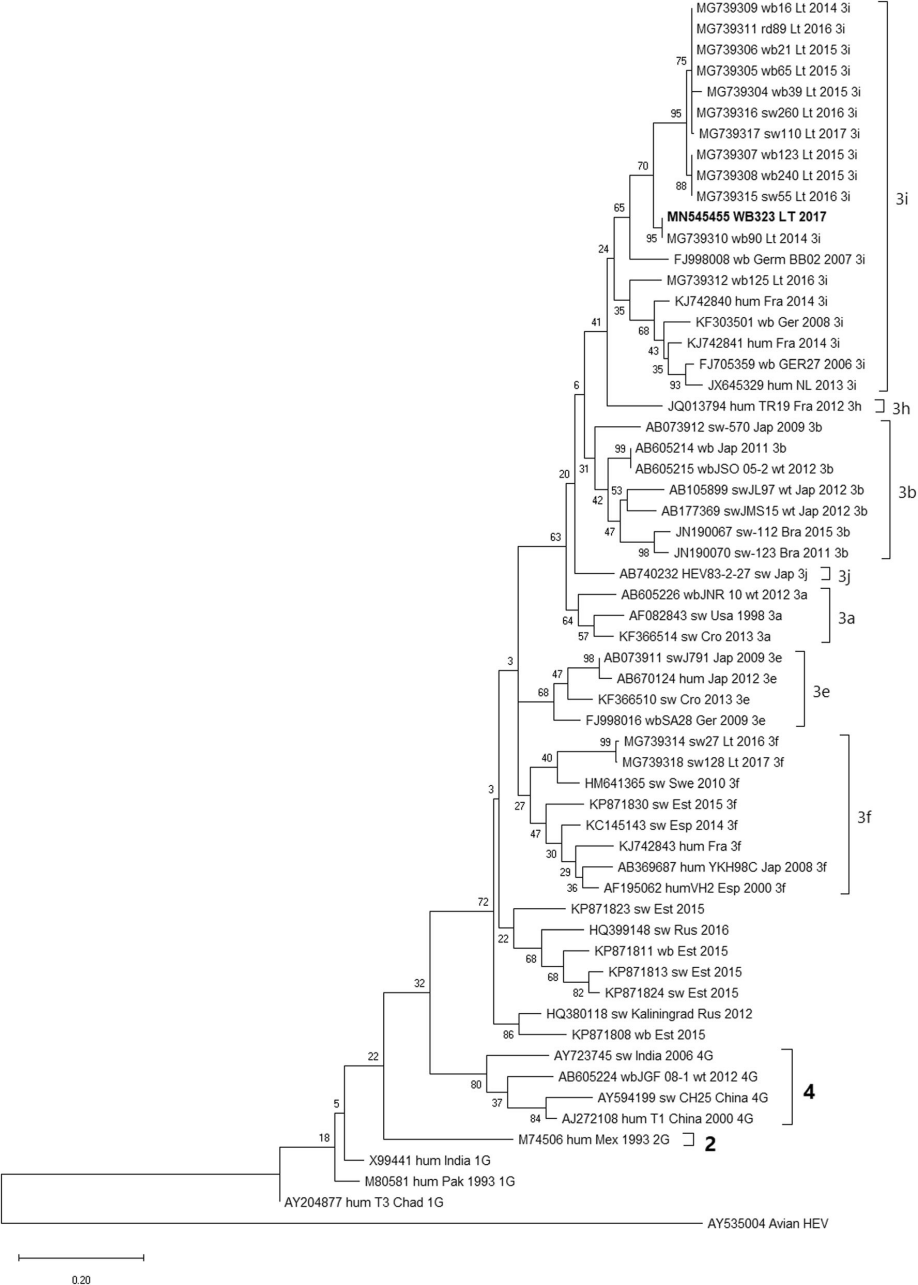
Phylogenetic tree constructed using neighbor-joining method. Comparison of partial ORF2 sequence of wild boar derived isolate (in bold), comprising 586 nt fragment, with 57 HEV reference sequences. Avian HEV (AY535004) was used as an outgroup. Reference sequences are denoted by respective accession numbers. Branch homology is indicated on each node by percentage of similarity. Used isolate WB323 is denoted in bold

Phylogenetic analysis based on a partial HEV ORF2 sequence showed the wild boar-derived virus clustering within 3i subtype of genotype 3 (Fig. 1), showing 95% homology with the nearest strain MG739310, also isolated from Lithuanian wild boar [15]. In addition, phylogenetic analysis of cell culture-generated virus isolates was performed after the final passage, showing 100% homology between wild type and cell culture-produced virus, confirming the successful propagation in tested cell lines.

### HEV presence in cell lines after infection without serial passaging

**Fig. 2:**
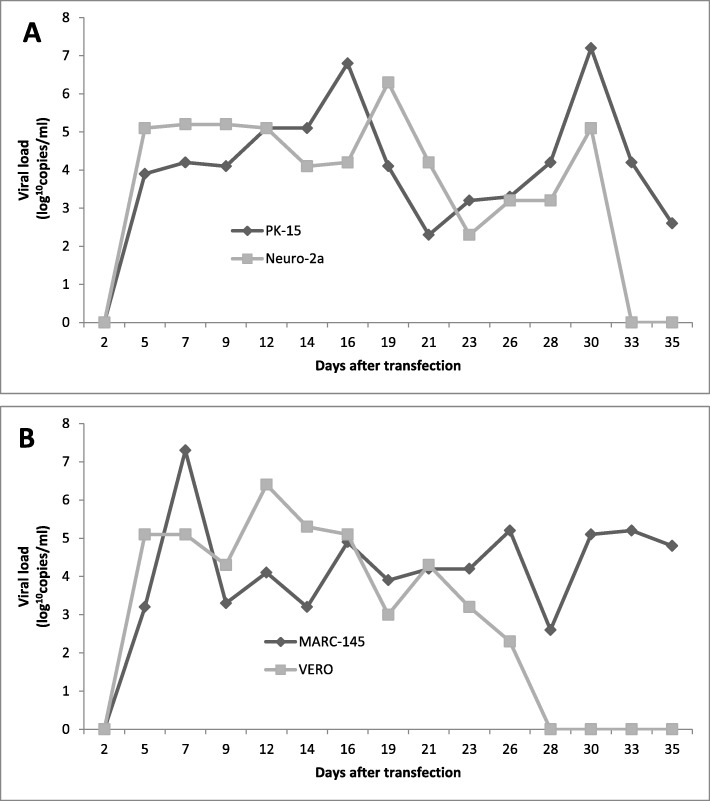
Viral load changes in culture mediums without serial passaging. **a** PK-15 and Neuro-2a cells, and (**b**) MARC-145 and VERO cell lines infected with HEV genotype 3 isolate from wild boar retained for indicated number of days

MARC-145, PK-15, Vero and Neuro-2a were successfully infected using wild boar-derived HEV strain. In all cell lines, HEV RNA was first detected in growth medium 5 days after inoculation, and remained present until day 33 and 28 in Neuro-2a and Vero cell lines respectively, and until day 35 in MARC-145 and PK-15 cell lines (Fig. 2). Changes in viral loads throughout the incubation period varied between cells, with peak loads observed on days 7, 12, 19 and 30 in MARC-145 (2.88 × 10^7^ copies/ml), Vero (4.23 × 10^6^ copies/ml), Neuro-2a (3.15 × 10^6^ copies/ml) and PK-15 (2.24 × 10^7^ copies/ml) cell lines respectively. Viral loads underwent similar fluctuations in PK-15 and Neuro-2a cells, with the initial peak appearing at days 16–19 post-inoculation (7.46 × 10^6^ and 3.15 × 10^6^ copies/ml in PK-15 and Neuro-2a cells, respectively), followed by a gradual decline and secondary peak at day 30 (2.24 × 10^7^ and 1.10 × 10^5^ copies/ml in PK-15 and Neuro-2a cells respectively) (Fig. 2a). No such two-phase change in viral loads was observed in MARC-145 and Vero cells (Fig. 2b).

### HEV presence in serial passages

After the initial seven-day incubation, the infected cells were frozen and thawed, HEV viral loads were determined and cell suspension was transferred on a fresh monolayer of cells. MARC-145 cells yielded the highest viral load (2.88 × 10^7^ copies/ml) after initial incubation (passage 0), which was consistent with the higher yield after the first passage (7.56 × 10^2^ copies/ml) compared to the other cell lines (Table 1). After the second passage, the highest RNA titers were detected in Neuro-2a (3.17 × 10^7^ copies/ml) and Vero (1.42 × 10^7^ copies/ml) cell lines. Similar RNA titers remained present after the third, fourth and fifth passages in both Neuro-2a and Vero cell lines. PK-15 cells showed a gradual growth in viral loads with every consecutive passage, reaching the final RNA titer of 2.38 × 10^5^ copies/ml after the fifth passage. Similarly, MARC-145 cells underwent a gradual growth in viral load and reached the final RNA titer of 1.12 × 10^7^ copies/ml.

**Table 1:** Viral load changes in cell culture medium after each serial passage

Passage	Days after initial inoculation	Viral load (copies/ml)			
		MARC-145	PK-15	Neuro-2a	Vero
0	7	2.88 × 10^7^	1.98 × 10^4^	1.88 × 10^5^	1.38 × 10^5^
1	14	7.56 × 10^2^	2.32 × 10^2^	2.45 × 10^2^	2.02 × 10^2^
2	21	9.17 × 10^4^	8.46 × 10^2^	3.17 × 10^7^	1.42 × 10^7^
3	28	9.41 × 10^6^	1.44 × 10^3^	5.05 × 10^7^	2.85 × 10^7^
4	35	1.84 × 10^6^	9.17 × 10^4^	1.90 × 10^7^	2.91 × 10^7^
5	42	1.12 × 10^7^	2.38 × 10^5^	2.97 × 10^7^	4.01 × 10^7^

### Differences between viral loads in trypsinised cells and culture medium of the neuro-2a line

**Fig. 3:**
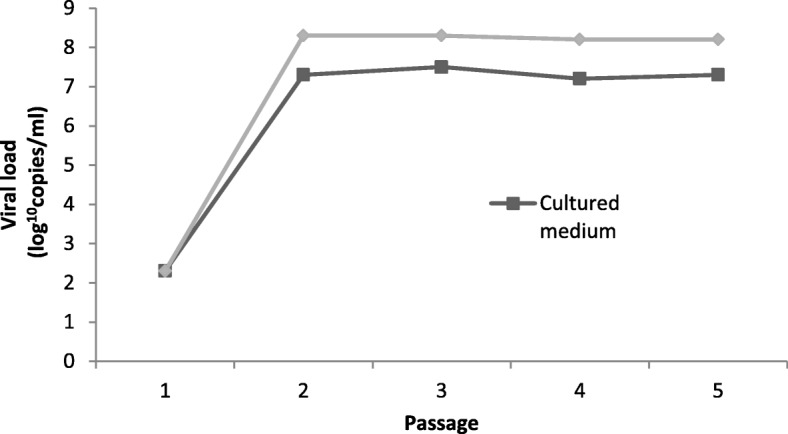
Difference in viral loads between cultured medium and trypsinized HEV infected Neuro-2a cells

Viral loads in the culture medium and cells harvested after each passage did not differ significantly in MARC-145, PK-15 and Vero cells (data not shown). However, a significant difference was detected in the Neuro-2a cells, where the viral loads in harvested cells reached 2.69 × 10^8^ and 2.83 × 10^8^ copies/ml compared to the cultured medium loads of 3.17 × 10^7^ and 5.05 × 10^7^ copies/ml after the second and third passages respectively (Fig. 3). The viral load difference of ~ 10^7^ compared to ~ 10^8^ copies/ml between cultured medium and trypsinised cells respectively was retained until the final passage.

### Immunofluorescence assay

**Fig. 4:**
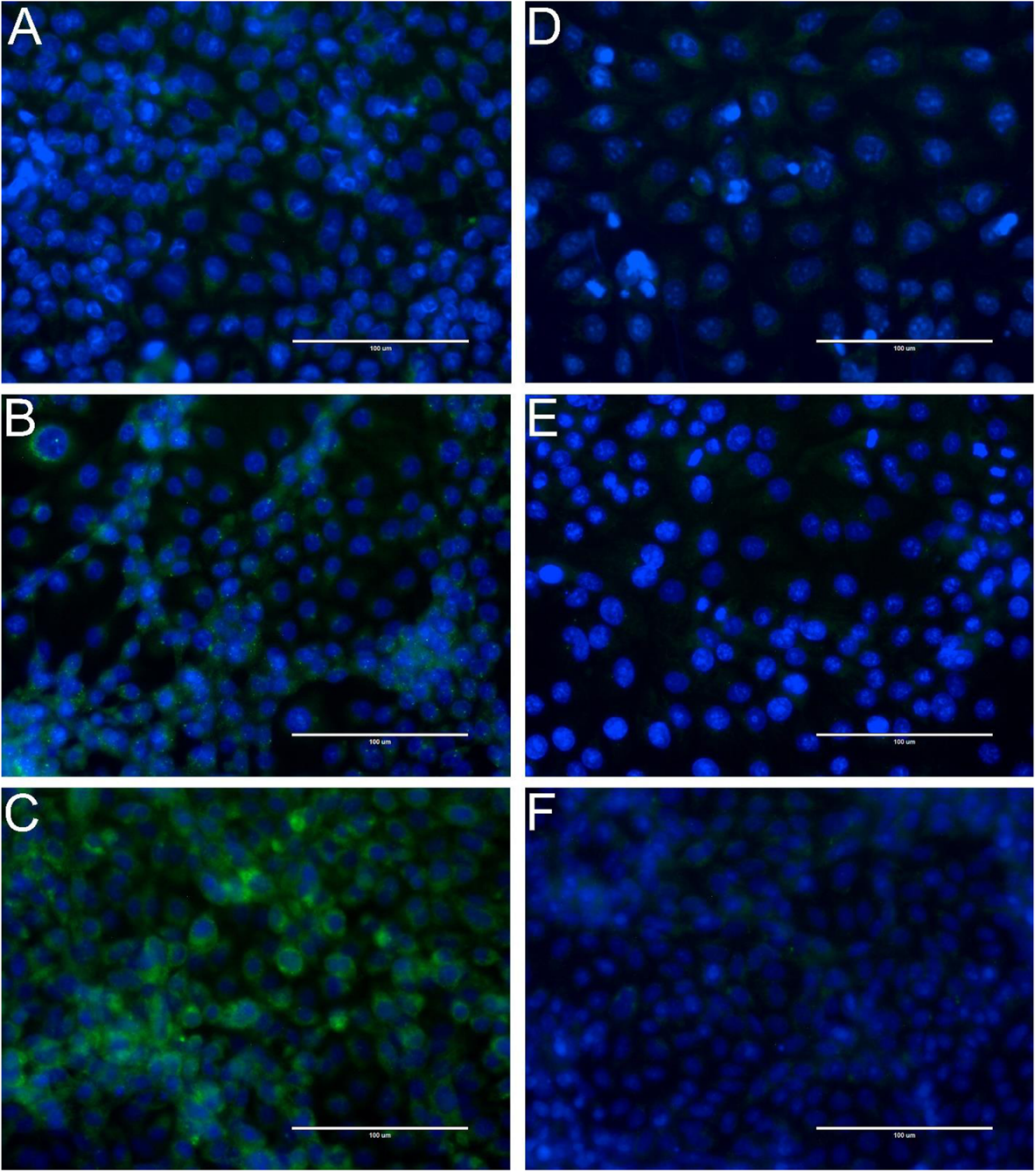
Immunofluorescence staining of HEV capsid protein in HEV-infected MARC, PK, and VERO cells. HEV capsid protein is green (Alexa Fluor 488 Plus or FITC), cell nuclei are blue (DAPI). Images of mAb 5F3-stained MARC-145 (**a**), PK-15 (**b**), Vero (**c**) cells and negative control staining of MARC-145 (**d**), PK-15 (**e**), Vero (**f**) cells. Scale bar: 100 μm

MARC-145, Vero, Neuro-2a and PK-15 cells inoculated with HEV strains were tested for the presence of HEV capsid protein by an immunofluorescence assay. Following MARC-145, Vero and PK-15 cell staining with HEV-3-specific mAb 5F3, a granular-like staining appeared, localised mainly in the cytoplasm of infected cells or groups of cells, and no staining was present in non-infected cells (Fig. 4). The observed staining of the HEV capsid protein indicates that a viral RNA has been synthesised as the HEV capsid protein is translated from a sub-genomic viral mRNA [16]. The detection of viral proteins confirmed that these cell lines were permissive for the tested HEV strain. Staining of Neuro-2a cells was unsuccessful as no specific fluorescence signal could be distinguished from a background (data not shown).

## Discussion

HEV isolation and replication in cell culture models is a complicated task. Considering the possible extra-hepatic localisation of the virus, cell lines of different origin have been reported as possible in vitro models. In particular, human lung cancer cell line A549 has been repeatedly demonstrated to be an efficient model for human-derived HEV genotypes 1, 3 and 4 isolation [17–20]. Non-human primate and porcine cells of kidney origin have been used for host range analysis of chimeric genotype 1–3 and genotype 1–4 constructs. Porcine kidney cells have proven to be particularly permissive for human-derived genotype 3 infections. However, it has been shown that genotype 1 strains (Sar-55 and Akluj) are also capable of infecting porcine kidney cells, although less efficiently [19], despite being associated with human infections only. Research of non-human-derived HEV strain isolation in cell lines of different origin has been limited. Porcine-derived HEV from liver samples and anal swabs have been shown to successfully infect porcine kidney cells [12]. Non-human primate kidney cells have not yet been used as a possible model for non-human-derived HEV isolation. Despite swine and wild boar-derived HEV strains sharing genetic similarities and often clustering along the same subtype, the host cell range of wild boar-derived HEV strains has not yet been determined. To date, only Takahashi et al. (2012) have used wild boar liver-derived HEV strains of genotypes 3 and 4 for successful human-derived cell line (A549 and PLC/PRF/5) infection [10]. However, wild boar-derived strains have not yet been used for experimentally infecting cell lines of non-human origin.

The present study shows that wild boar-derived HEV genotype 3 subtype 3i strain can efficiently replicate in non-human primate and porcine kidney cells and mouse neuroblastoma cells, with Neuro-2a and Vero cells capable of yielding viral loads of up to ~ 5 × 10^7^ copies/ml in serial passages. Interestingly, despite lower overall viral loads in serial passages of MARC-145 and PK-15 cells, the consistent presence of viral RNA in the aforementioned cells was detected for 35 days post-infection (Fig. 2) in contrast to Neuro-2a and Vero cells where HEV was no longer detectable after 30 and 26 days post-infection respectively. However, Neuro-2a and Vero cells consistently yielded HEV RNA after each serial passage, with samples from passage 6 being collected 42 days after initial inoculation. All the cell lines were inoculated with 2.75 × 10^7^ copies/ml of viral suspension. The viral loads were very similar in all infected cell lines after passage 1, except for MARC-145 where a marginally higher load was observed (7.56 × 10^2^ copies/ml in MARC-145 cells compared to ~ 2 × 10^2^ copies/ml in PK-15, Neuro-2a and Vero cells) (Table 1). In contrast to previous findings, it was discovered that a viral load of ~ 10^2^ copies/ml in the previous passage was sufficient to successfully infect a fresh monolayer of all the tested cell lines, as opposed to the proposed ~ 10^4^ copies per well by Tanaka et al. (2007) [7]. In the present study, total viral loads were determined by ORF2 detection using qRT-PCR; however, infectious virus loads were not determined separately, which could explain the possibility of lower total viral titer infecting fresh cell monolayers. Both in the present study and in Tanaka et al. (2007), ORF2 capsid protein production was not quantified. It has previously been demonstrated that inhibition of ORF2 translation in the host cells compromises the virion assembly and consecutive infection of other cells [19]. This allows speculation that the host cell qualities and characteristics of a particular genotype, specifically the ability of uninhibited translation of ORF2 capsid protein and formation of virions, and not viral load are responsible for successful infection of host cells. Higher viral loads might provide a greater probability of the presence of quasispecies harbouring mutations necessary for cultured cell infections. In the present study, high viral loads of initial infection suspension might have contained variants with sufficient genomic characteristics to ensure successful entry and replication in passage 0, which were then selected and multiplied in the consecutive passages, despite passage 1 yielding viral titer of < 10^4^ copies/ml.

Recent studies have concluded that HEV in cell culture suspension and blood is quasi-enveloped in a membrane of host cell origin [21–23]. Non-enveloped HEV is found in bile and faeces and within infected cells [24, 25]. It has been suggested that bile degrades the virus lipid quasi-envelope, resulting in non-enveloped HEV localisation in faeces. It is known that non-enveloped HEV and quasi-enveloped HEV (eHEV) have distinct entry mechanisms, and cells are less permissive to eHEV than to non-enveloped HEV [25]. In vitro [10, 25] and in vivo [26] research shows that the infectivity rate of faeces and liver-derived HEV is higher than serum (or EDTA plasma) and cell culture-derived HEV. The present data demonstrate that MARC-145 cells were infected more efficiently with liver-derived HEV (2.88 × 10^7^ copies/ml 7 dpi) compared to cell culture-derived HEV (7.56 × 10^2^ copies/ml 7 dpi) when infected with similar viral loads (2.75 × 10^7^ and 2.88 × 10^7^ copies/ml respectively). It is proposed that a higher load of non-enveloped HEV present in the infection mixture prepared from liver tissue resulted in higher viral loads after passage 0 in MARC-145 cells, whereas the mixture prepared from the previous passage and possibly harbouring higher loads of eHEV resulted in lower viral loads after passage 1. It is possible that the capability of non-enveloped HEV to effectively infect a range of different cells might be associated with a lack of immune response in the in vitro setting, as opposed to natural infection where the lipid coat is necessary for HEV to avoid neutralising antibodies, enabling extrahepatic localisation of eHEV.

Non-human primates such as cynomolgus macaques and rhesus macaques have previously been used as experimental models for human and swine-derived HEV infections [27, 28]. In the present study, both African green monkey (MARC-145) and vervet monkey (Vero) kidney cell lines proved to be permissive to the tested HEV strain, supporting cross-species transmission capabilities of genotype 3 between wild or domestic swine and non-human primates. Similarly in Takahashi et al. (2012), HEV isolated from wild boar liver homogenates was capable of infecting human A549 and PLC/PRF/5 cells, demonstrating HEV cross-species transmission between wild boar and primate cells [10]. The present study may be the first to demonstrate the permissiveness of primate-derived kidney cells to HEV genotype 3 isolated from wild boar.

HEV transmission between wild boars and domestic pigs has previously been demonstrated in experimental procedures [2]. In addition, Schlosser et al. (2014) report extra-hepatic localisation of HEV in experimentally infected pigs by detecting viral RNA in a variety of organs, including intestines, kidneys and spleen, depending on the infection entry route and suspension source. Extra-hepatic localisation of wild boar-derived HEV in infected pigs is consistent with the present findings concerning the permissiveness of porcine kidney cells (PK-15) to HEV isolated from wild boar. Porcine kidney cells, including the PK-15 line, have previously been used for the successful propagation of HEV genotype 4 and genotype 1–4 chimeras isolated from swine and humans respectively [12, 29]. However, the present study may be the first to demonstrate the capacity of swine kidney cells to be infected by wild boar-derived HEV genotype 3. Together with findings by Schlosser et al. (2014) and genetic analysis of HEV strains prevalent in wild boar and pig populations in Europe sharing up to 98% homology [2, 30], the present data are in support of the capability of HEV genotype 3 to cross the species barrier between wild boar and domestic pig populations, both of which are considered to be reservoirs for human infections.

Extra-hepatic localisation of HEV extends to nervous tissue and is often associated with clinical expression such as inflammatory polyradiculopathy, Guillain-Barré syndrome and neuralgic amyotrophy [31, 32]. There is currently a lack of data on the neurological symptom manifestation of HEV in animals. However, experimental infections of mice, macaque monkeys and rabbits have shown the presence of HEV in nervous tissue [14, 33]. Human cell lines of neural origin have proven to be particularly permissive to human-derived Kernow-C1p6 strain, where neuroblastoma SH-SH5Y and neural progenitor hES-NPCs cells showed HEV RNA levels higher than those detected in human hepatoma cells (HuH7) after infections [14]. In addition, primary cerebellar and hippocampal neurons extracted from mouse embryos have yielded significantly higher levels of HEV compared to HuH7 cells after inoculation. The present study demonstrated similar findings in mouse neuroblastoma (Neuro-2a) cells, where peak concentrations of HEV RNA were detected 19 and 30 days after infection, and consistent viral loads of ~ 10^7^ copies/ml were reached after passage 2 onwards. The ability of a wild HEV strain to infect neural cells of mouse origin is also compatible with HEV genotype 3 capabilities to cross the species barrier, suggesting a possible capacity for clinical HEV expression in animal populations. Unsuccessful staining of NBL cells using mAb 5F3 conjugated with FITC could be due to the murine origin of the cell line. Staining of murine cells with murine antibodies often gives a high background in the fluorescence signal. The specific fluorescence signal of HEV localisation in Neuro-2a cells could have been suppressed by the high background signal observed in HEV-inoculated cells and non-inoculated cells.

Interestingly, Neuro-2a was the only tested cell line in which a significant difference between HEV viral loads in culture medium and trypsinised cells was observed. No significant difference in HEV RNA levels was observed in the culture medium and trypsinised cells of MARC-145, PK-15 and Vero lines, possibly indicating unrestricted ORF2 production, virion assembly and release. Higher viral loads in trypsinised Neuro-2a cells indicate possible restrictions to successful viral release. In turn, elimination of speculated restrictions associated with host cells could result in even higher yields of HEV in Neuro-2a culture medium, consistent with previous findings about the efficiency of neuroblastoma cells as in vitro models [14]. A wider range of HEV genotypes should be tested on Neuro-2a cells to determine the consistency of these findings and discriminate between cell host and genotypic characteristics of the virus as possible causes for restricted egress.

Although HEV proves difficult to isolate in cell cultures, particular strains and possibly quasispecies are capable of propagating in a range of cell lines of different animal and tissue origin. Kernow-C1 strain has proven to be remarkably viable, and both ORF2 and ORF3 proteins have been detected not only in different human, porcine kidney and rhesus macaque kidney cells, but also in cow, mouse, chicken, cat, dog and rabbit cells [19]. The HEV strain of wild boar origin proved to be similarly versatile and capable of entry, propagation and release from porcine and monkey kidney cells, in addition to mouse neural cells. However, the genetic identity of WB323 partial ORF2 region to other genotype 3 strains of similar origin or capacity to infect a variety of cell lines did not exceed 90%, indicating that different genotype 3 subtypes could be capable of wider cellular tropism (Fig. 1). Full HEV isolate genome sequencing would be necessary to conduct a thorough comparison of the aforementioned strains and identify similarities in significant segments of the HEV genome, considering that 586 nt and 363 nt portions of ORF2 chosen in this study are highly conservative among different HEV genotypes.

Several limitations of this study need to be addressed. The lack of data from the immunofluorescence assay about the presence of HEV capsid protein in infected Neuro-2a cells refutes an important piece of evidence supporting HEV replication in Neuro-2a cells. Measurements of HEV RNA in Neuro-2a cells by qRT-PCR could be supplemented by investigating the capacity of progeny virus from Neuro-2a cells to infect other viable cell lines. In the present study, only one genotype 3 strain isolated from the Lithuanian wild boar population was tested. In order to further confirm the viability of tested cell lines for wild boar-derived HEV genotype 3 isolation, more closely related HEV strains should be tested. In addition, already established cell lines (e.g. A549, PLC/PRF/5) were not used alongside PK-15, MARC-145, Vero and Neuro-2a cell lines for experimental infections, making it difficult to compare their viability. Taq-man real-time RT-PCR protocol is primarily used for genome equivalent quantification with the ORF3 fragment of HEV [34], however the SYBR Green real-time RT-PCR protocol was chosen for the present study because it proved to be a better choice when using ORF2 for plasmid construction and genome equivalent calculations than the Taq-man real-time RT-PCR protocol, which was found to be less reliable for standard curve construction.

## Conclusions

This study shows for the first time that wild boar-derived HEV genotype 3 subtype 3i strain was capable of infecting cell lines of animal origin, including primate and porcine kidney cells (MARC-145, PK-15 and Vero), and mouse neuroblastoma cells (Neuro-2a), supporting the notion of the capacity of HEV genotype 3 to cross the species barrier and extra-hepatic localisation of the virus. Higher yields of HEV RNA in Neuro-2a cells compared to the rest of tested cell lines proved the capacity of neural cells to harbour HEV and serve as an efficient in vitro model for viral isolation and amplification. Therefore, the present study warrants further studies of tested cell lines as a possible efficient means of HEV genotype 3 propagation. HEV isolates from other wild animal hosts, such as roe deer and moose, should be isolated on selected cell lines to shed more light on HEV transmission dynamics between wild animal populations and their role as sources of zoonotic infections.

## Methods

### Virus strain

HEV isolate was obtained from a liver sample of HEV RNA-positive wild boar (data not shown) hunted in Lithuania (Elektrenai municipality) in 2017 (WB323) and collected by a state veterinarian as part of a national infectious disease surveillance programme. The liver tissue sample was collected during the dressing of the carcass and stored at − 20 °C for further analysis. The frozen sample was scraped with a razor blade, homogenised in PBS (1x, pH 7.2; Gibco), and centrifuged at 3000 g and 12,000 g for 10 and 5 min respectively until a clear supernatant was acquired and passed through a 0.22-μm pore size microfilter (Techno Plastic Products) for purification. The HEV RNA load of the virus stock was determined to be 2.75 × 10^7^ copies/ml.

### Cell lines

The following cell lines were acquired: pig kidney cells (PK-15 ATCC No. CCL-33), monkey kidney cells (MARC-145 ATCC No. CRL-12231; Vero ATCC No. CCL-81) and murine neuroblastoma cells (Neuro-2a ATCC No. CCL-131). All the cell lines, except for Neuro-2a, were cultured in Minimum Essential Medium (MEM; Gibco) with additional 10% heat-inactivated fetal bovine serum (FBS; Gibco), 100 U/ml penicillin and 100 μg/ml streptomycin. Neuro-2a cells were cultured in Dulbecco’s modified Eagle’s medium (DMEM; Gibco) with additional 10% FBS, 100 U/ml penicillin and 100 μg/ml streptomycin. Cells were grown at 37 °C. Monolayers of cells were trypsinised, diluted (1:3 and 1:6 dilutions for Neuro-2a and PK-15 cells respectively, and 1:4 for MARC-145 and Vero cell lines) in growth medium and transferred to 25-cm^2^ tissue culture flasks (Techno Plastic Products) 1 day before virus inoculation.

### Cell line infection and serial passage

Growth medium was removed from the flasks with cell monolayers, washed with 5 ml PBS and inoculated with 1 ml purified virus stock. The infection mixture was replaced 1 h after incubation at room temperature with 10 ml maintenance medium, consisting of MEM (DMEM for Neuro-2a cells) containing 10% FBS, 100 U/ml penicillin and 100 μg/ml streptomycin. Further growth of infected cells was carried out at 37 °C. Until the end of experiment, 5 ml growth medium was collected from culture flasks every two to 3 days and stored for further analysis, replaced by 5 ml fresh maintenance medium. At 35 days after infection, the cells were trypsinised and centrifuged for 5 min at 3000 g, the supernatant was removed and the cells were sedimented and stored in addition to the growth medium. All infections were performed in triplicate sets.

Alongside the retention of HEV-infected cell lines for 35 days, a serial passage experiment using PK-15, MARC-145, Vero and Neuro-2a cell lines was also performed. Before each serial passage, infected cells were frozen and thawed three times to prepare an infection mixture from the previous passage. The maintenance medium of the fresh cell monolayer was replaced with 1 ml infection mixture and incubated at room temperature for 1 hour, after which the solution was removed and replaced by 10 ml maintenance medium. Further incubation was carried out under the conditions described above. All serial passages were performed in triplicate sets. After each passage, growth medium and trypsinised cell samples were collected and tested separately. In addition, sequencing of the ORF2 region of HEV RNA from the final passage was performed to validate the presence of the selected HEV strain.

### Quantification of viral RNA

Total RNA was extracted from cell and growth medium samples using the GeneJET RNA Purification Kit (Thermo Scientific) according to the manufacturer’s instructions. Primers targeting the ORF2 region of HEV RNA were chosen and quantitative HEV RNA real-time PCR (qRT-PCR) was performed according to the modified method described previously [34]. Briefly, plasmid pJET1.2 was constructed from the wild boar-derived HEV strain described above using the CloneJET PCR Cloning Kit (Thermo Scientific) and TransformAid Bacterial Transformation Kit (Thermo Scientific) according to the manufacturer’s instructions. Plasmid DNA was extracted using the GeneJET Plasmid Miniprep Kit (Thermo Scientific) and quantified using the Qubit dsDNA BR Assay Kit (Invitrogen) according to the manufacturers’ instructions. Standard curves were generated after 10-fold dilutions of stock DNA, resulting in viral copy equivalents ranging from 10^2^ to 10^8^. For cDNA synthesis, Oligo (dT)18 Primer (Thermo Scientific) with reverse transcriptase was used according to the manufacturer’s instructions. Real-time PCR was performed using SYBR Green I dye (Thermo Scientific) and primer sets described in Table 2, generating 347 bp product [35]. Reverse transcription was carried out at 42 °C for 60 min and an additional 70 °C step for 10 min. The quantitative PCR thermal cycling conditions were polymerase activation at 95 °C for 10 min, 40 cycles of 94 °C for 1 min, 55 °C for 1 min and 72 °C for 1 min, before a final extension at 72 °C for 1 min. All the samples were tested in triplicates and mean values of viral copy equivalents were calculated.

**Table 2:** Primer sets used in the study

Sequence (5` → 3`)	Role	Product length (nt)	Target position	Reference
GTWATGCTYTGCATWCATGGCT	qRT-PCR	347	5972–5993	[35]
AGCCGACGAAATCAATTCTGTC			6298–6319	
AATTATGCYCAGTAYCGRGTTG	Sequencing	586	5687–5708	[35]
CCCTTRTCYTGCTGMGCATTCTC			6395–6417	

### Sequencing and phylogenetic analysis

For further genetic characterisation of wild boar-derived HEV strain, the nucleotide sequence was acquired using the primer set described in Table 2 and submitted to GenBank (Accession number MN545455. A partial ORF2 sequence comprising a 586 nt long sequence of the wild boar-derived HEV strain was compared to selected HEV genotype 3 strains isolated from human, swine and wild boar hosts. Multiple alignment of all sequences was created using ClustalW software in MEGA X package [36]. The neighbour-joining method was used for phylogenetic tree construction with 1000 bootstrapping replicates.

### Immunofluorescence assay

Infected MARC-145, PK-15, Vero and Neuro-2a cells grown on 10-cm^2^ tissue culture flasks (Clipmax, TPP) were used for immunofluorescence assay after 14 dpi. On day 14, cell culture media were removed, the cells were washed with PBS and fixed using ice-cold methanol (Roth) and acetone (Roth) mixture in equal parts 1 ml/slide for 5 min at − 20 °C. The slides were then either air-dried and stored at 4 °C until use or washed in PBS and blocked for 1 h at room temperature with 10% Normal Goat Serum (Invitrogen) for MARC-145, Vero and PK-15 cells and with 20 μg/ml polyclonal antibodies from a non-immunised mouse diluted in 10% Normal Goat Serum for Neuro-2a cells to block Fc receptors as the cells were of murine origin. HEV capsid proteins were stained using HEV genotype 3 capsid protein-specific monoclonal antibody clone 5F3 (mAb 5F3) [37]. For MARC-145, Vero and PK-15 cells, mAb 5F3 was diluted to a concentration of 10 μg/ml in PBS containing 3% bovine serum albumin (BSA, GE Healthcare) and incubated for 1 h at room temperature. The mAbs were detected by Alexa Fluor 488 Plus-conjugated Highly Cross-Adsorbed Goat anti-Mouse IgG Antibodies (Invitrogen) diluted 1:200 in PBS with 3% BSA and incubated for 1 h at room temperature. For Neuro-2a cells, fluorescein isothiocyanate (FITC, Sigma-Aldrich)-conjugated mAb 5F3 diluted in PBS with 3% BSA was used. mAb 5F3 conjugation with FITC was performed according to the manufacturer’s instructions. After washing with PBS, the slides were mounted using ProLong™ Diamond Antifade Mountant with 4′,6-diamidino-2-phenylindole (DAPI) (Invitrogen). The stained cells were imaged using EVOS FL Auto Imaging System (Life Technologies).

## Data Availability

The nucleotide sequence of HEV generated and used during the current study has been assigned DDBJ/EMBL/GenBank Accession number MN545455.

## Abbreviations

BSA: Bovine serum albumin
cDNA: complementary DNA
DAPI: 4′,6-diamidino-2-phenylindole
DMEM: Dulbecco’s modified Eagle’s medium
DNA: Deoxyribonucleic acid
dpi: days post-infection
EDTA: Ethylenediaminetetraacetic acid
eHEV: quasi-enveloped HEV
FBS: Fetal bovine serum
FITC: Fluorescein isothiocyanate
HEV: Hepatitis E virus
mAb: monoclonal antibody
MEM: Minimum essential medium
mRNA: messenger RNA
ORF: Open reading frame
PBS: Phosphate-buffered saline
qRT-PCR: real-time reverse transcription polymerase chain reaction
RNA: Ribonucleic acid
